# Effectiveness and safety of acupuncture for insomnia: Protocol for a systematic review

**DOI:** 10.1097/MD.0000000000017842

**Published:** 2019-11-11

**Authors:** Mingming Zhang, Jingwei Zhao, Xiao Li, Xinwang Chen, Jin Xie, Lingyan Meng, Xiyan Gao

**Affiliations:** a Department of acupuncture and moxibustion, Henan University of Chinese Medicine; b Department of geriatrics, The First Affiliated Hospital of Henan University of Chinese Medicine; c Department of acupuncture and moxibustion, The Third Affiliated Hospital of Henan University of Chinese Medicine, Zhengzhou, China.

**Keywords:** acupuncture, insomnia, systematic review, treatment program

## Abstract

**Background:**

Insomnia is one of the most common diseases in modern society, the main characteristics of the patients were difficulty in falling asleep at night and/or failure to maintain effective sleep after falling asleep. It can lead to early awakening, short sleep, heavy sleeplessness, dreaming, poor sleep quality, and working hours after waking up, causes a series of negative emotions, such as fatigue, inefficiency, cognitive decline, social interaction, tension, and anxiety, which affect social harmony and stability. So Insomnia has gained more and more attention. At present, acupuncture has been proved effective in the treatment of insomnia by many studies. The purpose of this study is to evaluate the efficacy and safety of acupuncture in the treatment of insomnia, and to provide the latest evidence for clinical application.

**Methods and analysis:**

We collected the qualified literature on acupuncture treatment of insomnia by electronic retrieval of Cochrane Library, China National Knowledge Infrastructure (CNKI), China Biomedical Disc (CBMDISC), PubMed, China Science and Technology Journal Database (VIP) and Wanfang Database, and manual retrieval of papers and internal reports. We will select the eligible studies published up to September 30, 2019. We use Insomnia Severity Index (ISI) as the main outcome of insomnia and Pittsburgh Sleep Quality Index (PSQI), Hamilton Depression Scale(HAMD) and Self-Rating Anxiety Scale (SAS) as secondary indicators to evaluate the efficacy and safety of acupuncture treatment of insomnia, we will use Revman v.5.3 software to calculate data synthesis, and if the results are appropriate, meta-analysis can also be carried out.

**Results:**

This study will provide comprehensive evidence of high quality of acupuncture treatment for insomnia from ISI, PSQI, HAMD, SAS, and adverse reactions.

**Conclusion:**

The systematic review will provide a basis for evaluating the efficacy and safety of acupuncture in the treatment of insomnia.

**Trial registration number:**

PROSPERO CRD42019131957.

## 1 Introduction

Insomnia is one of the most common diseases in modern society, around 15% to 30% of adults and 10% to 23% of adolescents worldwide have different degrees of insomnia.^[1,2]^ It can lead to early awakening, short sleep, heavy sleeplessness, dreaming, poor sleep quality, and working hours after waking up, causes a series of negative emotions, such as fatigue, inefficiency, cognitive decline, social interaction, tension, and anxiety, which affect social harmony and stability. So Insomnia has gained more and more attention.^[3,4]^

Currently, because insomnia is a subjective complaint, we have not yet reached a consensus on the causes of insomnia, so it is difficult to define and diagnose.^[5]^ Summers et al defines insomnia by its symptoms as complaints of sleep disturbance, such as difficulty in falling asleep or disturbance of sleep maintenance, and/or early awakening.^[6]^ At the same time, Doghramji adds daytime injuries to supplement, such as anxiety, fatigue, retardation, memory loss, and general discomfort affecting many aspects of daytime activity.^[7]^

At present, there are 3 international diagnostic criteria for insomnia, The Diagnostic and Statistical Manual of Mental Disorders (DSM), The International Classification of Sleep Disorders, and The ICD-10 Classification of Mental and Behavioral Disorders. The European Sleep Research Society puts forward 3 diagnostic criteria for insomnia:

1. the presence of night sleep disorders (difficulty in falling asleep, maintenance disorders, early awakening, etc.),
2. related daytime impairments (fatigue, memory loss, inattention, etc.),
3. sleep disorders occur at least 3 nights a week, and persistence Continue for 3 months or more.^[8]^

Assessment of insomnia is mainly based on a history of syndromes and screening for complications such as depression and anxiety, respiratory diseases, and drug use.^[9]^ Many of the tools used to assess insomnia are subjective questionnaires. Including Pittsburgh Sleep Quality Index (PSQI), Insomnia Severity Index (ISI), etc. Other ways include sleep logs, symptom checklists, psychological screening tests, and bed companion interviews. The Pittsburgh Sleep Quality Index is a sleep questionnaire that provides useful information about sleep quality, time, and duration.^[10]^ Insomnia severity index (ISI) has been proved to be a reliable and effective tool for evaluating the severity of insomnia.^[11]^ In addition, if necessary, the use of activity recorder or polysomnography should be considered.

At present, the treatment of insomnia mainly includes cognitive behavioral therapy and other psychotherapy,^[12]^ oral drug therapy,^[13]^ phototherapy^[14]^ and exercise therapy,^[15]^ complementary and alternative medical therapy. Supplementary and alternative medical therapies have proposed several treatments for insomnia, including acupuncture, acupoint massage, aromatherapy, foot reflex therapy, homeopathy, meditation exercise therapy, moxibustion, music therapy, and yoga. Acupuncture, as one of the complementary and alternative medical therapies for insomnia, has been proved to be an effective method for insomnia in many clinical trials.^[16–19]^ However, its efficacy needs further evaluation. In addition, no publication of similar system reviews has been retrieved in the database. Therefore, this article hopes to evaluate the efficacy and safety of acupuncture in the treatment of insomnia, and provide new evidence for clinical application.

## 2 Methods

The protocol has been registered on PROSPERO as CRD42019131957. (https://www.crd.york.ac.uk/prospero/display_record.php?RecordID=131957). The protocol follows the Preferred Reporting Items for Systematic Reviews and Meta-Analyses Protocols (PRISMA-P) statement guidelines. We will describe the changes in the full review if necessary.

### 2.1 Inclusion criteria for study selection

#### 2.1.1 Types of studies

This study will be included in a randomized controlled trial (RCT) of acupuncture in the treatment of insomnia, whether using blind method or allocation concealment method. The language of the trial is limited to Chinese and English. Non-randomized controlled trials, a series of case reports, and cross-over studies were excluded.

#### 2.1.2 Types of participants

Regardless of the subtype of insomnia, all participants diagnosed with insomnia will receive attention. There will be no restrictions on gender, age, race, economic status, or education.

#### 2.1.3 Types of interventions

The treatment group was treated with acupuncture, acupoints, and frequency were not limited. At the same time, the control group was intervened with placebo.

#### 2.1.4 Types of outcome measures

##### 2.1.4.1 Primary outcomes

We use ISI as the primary outcome of insomnia.

##### 2.1.4.2 Secondary outcomes

We also care about the following indexes: PSQI; HAMD; SAS. In addition, we will carefully observe the adverse reactions of patients during acupuncture.

### 2.2 Search methods for the identification of studies

#### 2.2.1 Electronic searches

Following databases will be searched: PubMed, MEDLINE, EMBASE, Cochrane Library, China National Knowledge Infrastructure (CNKI), Wanfang data, Chinese Scientific Journals Database (VIP), and China biomedical literature database(CBM). We will select the eligible studies published up to September 30, 2019. The search terms used in the systematic review are as follows: acupuncture, insomnia, Sleep disorder, budewo, budemian, and budeming. We will not apply any language, population or national restrictions.

The specific search strategy will be (taking PubMed as an example):

Search ((((Insomnia[Title/Abstract] OR Sleep disorder[Title/Abstract]))) AND (Acupuncture[Title/Abstract])), And similar search strategy will be applied to other electronic databases.

#### 2.2.2 Searching other resources

We also retrieve manual related documents, such as replacing and supplementing some reference documents, such as medical textbooks and clinical laboratory manuals, and the World Health Organization International Registry of Clinical Trials (ICTRP). At the same time, we will contact experts and authors in this field to obtain important information that cannot be found in the search.

### 2.3 Data collection and analysis

#### 2.3.1 Selection of studies

**Figure 1:**
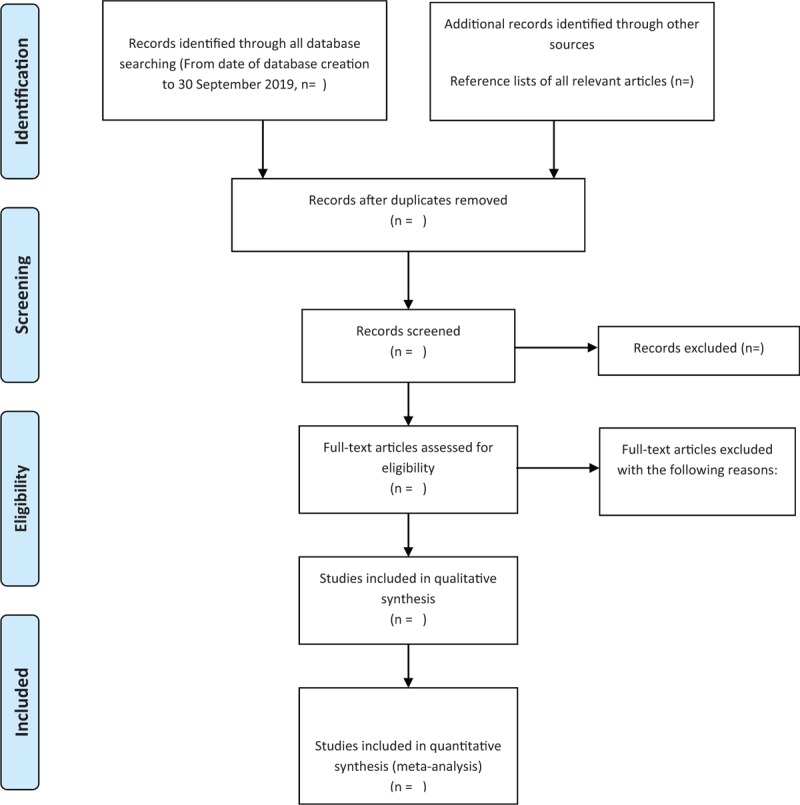
The PRISMA flow chart. Flow diagram of study selection process.

Two experienced reviewers independently searched all databases, excluded obviously unrelated literature, and deleted duplicated relevant research abstracts, then read the remaining abstracts and the full text, and screened the full text according to the previous contents to determine the criteria for qualified research. If the information is incomplete, the reviewer should contact the author to obtain complete information and delete the full text of the research review will record the specific reasons for exclusion. If there is a disagreement, it will be resolved through a panel discussion. Another reviewer will review the selection process. Details of the selection process will be shown in the Prisma flowchart (Fig. 1).

#### 2.3.2 Data extraction and management

Two reviewers will independently retrieve data based on a pre-designed data collection form and use spreadsheets to extract the following information: research details (first author's name or communication, publication time, publication year, multi-center study, or non-research), participant details (baseline data, diagnostic criteria for insomnia, exclusion criteria, gender, year) Age, methods used (sample size, blindness, distribution concealment), interventions in treatment group and control group, major and minor outcomes (ISI, PSQI, HAMD, SAS, follow-up, adverse events). If the above data display is incomplete, we will contact the author of the article. If necessary, all reviewers participate in discussions to resolve differences.

#### 2.3.3 Assessment of risk of bias in included studies

Two reviewers will evaluate the quality of each article using Cochrane Collaboration Tool to assess bias risk.^[20]^ The review includes generating random sequences, allocating concealment, deceiving participants and personnel, incomplete results data, selective reporting, and other sources of bias. According to the criteria, it is classified as low risk, high risk, and unclear. Two reviewers will be independently involved in the evaluation of each study, and any differences will be resolved through discussion.

#### 2.3.4 Measures of treatment effect

We will record the mean difference (MD) or standardized mean difference (SMD) and 95% confidence interval (CI) for continuous variable outcomes. While for dichotomous outcomes, we will record the relative risk (RR) and 95% CI.

#### 2.3.5 Dealing with missing data

Auditors collect events in clinical reports that are unclear or do not report data and send them to the authors of clinical reports by telephone, fax, and e-mail at the first time.

#### 2.3.6 Assessment of heterogeneity

All literature will use *I*^2^ value of the Chi-Squared test (α = 0.1) to determine the heterogeneity. When *I*^2^ ≤ 50%, it is considered acceptable. When *I*^2^ > 50%, subgroup analysis should be performed to identify potential causes and record them.

#### 2.3.7 Data synthesis and analysis

Data synthesis and analysis will use Revman V.5.3.5. When the heterogeneity of statistical descriptions is not obvious, the reviewer implements a fixed effect model. On the contrary, the random effect model was used to detect the sources of statistical heterogeneity. If heterogeneity is significant, researchers can turn to subgroups or sensitivity analysis, α = 0.05 for evaluating meta-analysis.

#### 2.3.8 Subgroup analysis

When meta-analysis shows significant heterogeneity, we will subgroup analysis according to the type of insomnia and different methods of acupuncture.

#### 2.3.9 Sensitivity analysis

Sensitivity analysis will be used to test the quality of the research contained in the sampled documents. The stability of the conclusions can be tested by re-analyzing the conclusions by inputting missing data and changing the type of research.

#### 2.3.10 Ethics and dissemination

The results of the system review will be published in peer-reviewed journals, disseminated at relevant meetings, or disseminated in peer-reviewed publications, and we use aggregated published data to exclude individual patient data, so ethical approval, and informed consent are not required.

## 3 Discussion

**Figure 2:**
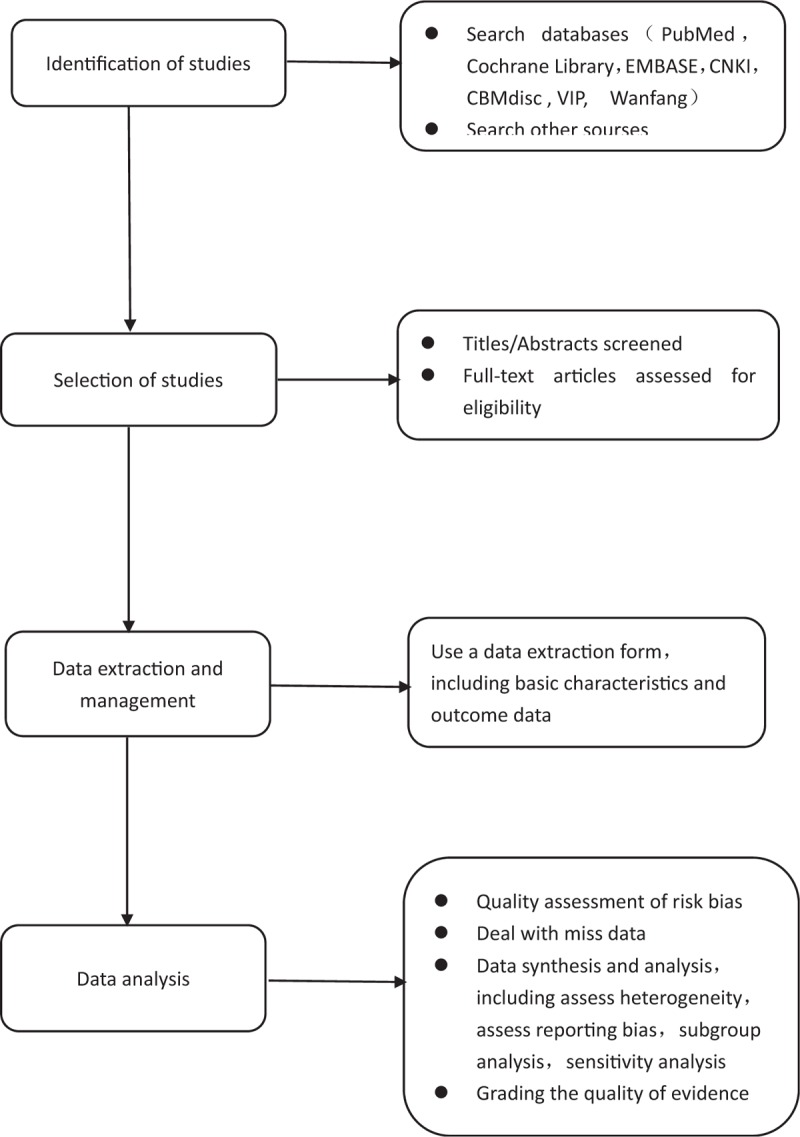
Flow diagram of the systematic review and meta-analysis.

Insomnia is one of the most common diseases in modern society. Almost everyone has experienced or is experiencing insomnia, which can lead to a series of negative emotions, such as fatigue, inefficiency, cognitive decline, social interaction, tension, and anxiety. It is also a risk factor of arterial hypertension, myocardial infarction, and chronic heart failure,^[21,22]^ which can give society and families. It brings great psychological and economic burden. Currently, the first-line drugs available for clinical treatment of insomnia include benzodiazepines(BZ) and benzodiazepine receptor agonists(BZRAs), antidepressants, antipsychotics, antihistamines, phytotherapeutic substances, and melatonin.^[23]^ However, long-term use of these drugs will bring some side effects to patients, such as headache, dizziness, dry mouth, abnormal taste. Acupuncture may be one of the alternative methods to treat insomnia, with little side effects. At present, there are many reports that acupuncture has a significant effect on insomnia, but the safety of acupuncture in the treatment of insomnia is uncertain. So far, no systematic review has been reported. Therefore, it is necessary to conduct a high-quality systematic review and meta-analysis to systematically evaluate the published randomized controlled trials of acupuncture and moxibustion for insomnia and to provide objective evidence for its clinical application. The process of performing this systematic review, shown in Figure 2, including identification of studies, selection of studies, data extraction and management, and data analysis.

However, there are still some limitations in this study. Firstly, due to the limitations of English and Chinese, there may be some risk of bias. Secondly, the heterogeneity of acupuncture may be significant due to the different methods of acupuncture. Finally, smaller sample size experiments will lead to higher risk bias.

## Author contributions

**Data curation:** Mingming Zhang, Jingwei Zhao.

**Formal analysis:** Mingming Zhang, Jingwei Zhao.

**Funding acquisition:** Xiyan Gao.

**Methodology:** Xiao Li.

**Project administration:** Xinwang Chen, Lingyan Meng.

**Software:** Xiao Li, Xinwang Chen.

**Supervision:** Xiyan Gao.

**Validation:** Xiyan Gao.

**Visualization:** Jin Xie.

**Writing – original draft:** Mingming Zhang, Xiyan Gao.

**Writing – review & editing:** Mingming Zhang, Xiyan Gao.
